# Mr. Litle on the Fatal Termination of Wounds in the Navy

**Published:** 1812-08

**Authors:** James Litle

**Affiliations:** Surgeon, His Majesty's Ship Salvador del Mundo, at Plymouth


THE
Medical and Physical Journal.

2 OF VOL. XXVIII.] AUGUST, 1812. [NO. 162.

*To the Editors of the Medical and Physical Journal.*

GENTLEMEN,

THROUGH the medium of your valuable publication, I beg leave to submit, for the consideration of my brethren in the navy, a few observations upon some of the practical occurrences which take place in the medical department, during, and immediately subsequent to, naval engagements at sea. Every subject connected with our naval prosperity acquires much importance, and few, perhaps, more than that which tends to give confidence to the minds of those valuable defenders of the state, in the assistance which awaits them, should they be wounded in battle. For seamen (though in other respects so inattentive to their own welfare) in no small degree appreciate the merit of their surgeon on such an occasion. A favorable impression ought, therefore, to be sedulously inculcated in their minds, as it tends not a little to inspire them with confidence, and spur them on to unusual exertion, when they have full confidence in the professional abilities and experience of the man whose hands they are to come under, should they stand in need of his aid.

During my professional career of service in the navy, I have had occasion to remark, and it has also been often mentioned by others of long experience, who have been much in action, that in many cases, when the amputation of a limb is immediately required to be performed, the patient has expired under the operation, or died soon after the dressing was completed; notwithstanding the operation was performed with all the science and regularity of the most experienced and able hand, and even though the subject had an excellent constitution, that no previous hæmorrhage had taken place, or any other untoward occurrence had intervened which could have led us to expect such a fatal event; yet it often happens, that, when a seaman is wounded, (we will say, for example, his leg taken off by a cannon-ball,) he is immediately brought down to the cockpit, and the limb is amputated; which, though performed with all requisite skill, yet many cases have occurred in this particular situation, where the patient has expired under the knife, or perished soon after the operation was completed, it being the case in amputations of the lower limbs more particularly. And perhaps the danger, in a certain ratio, is commensurate with the extent of substance removed, or disorganisation of parts connected with the wound; for I cannot say from experience that it often occurs from punctured or musket-ball wounds, but every surgeon in the navy, who has been much in action, and consequently conversant with the destructive occurrences which ensue on such an occasion, must have met with many melancholy examples of the kind, though perhaps, in the general bustle and confusion which such a scene exhibits, events at that particular crisis might have been omitted to be recorded, or considered with that attention which so serious a subject requires; but with us, particularly in the navy, it is an incumbent duty to narrate even such cases, as it would lead to examination, and perhaps eventually to a more successful method of treatment. And, when we consider the naval force of our enemy, which is so fast increasing to a gigantic magnitude, single and general battles, we may reasonably infer, will ere long accumulate fast upon us. The question, therefore, opens an important point of practice, to which I earnestly request to call the attention of the medical officers of the navy, as it involves a fact of the deepest importance.

For example, in that most severe conflict which has recently taken place between the Victorious and the Rivoli, we read in the newspapers, in the list of killed and wounded, (which appears to be detailed with much care by the surgeon of the Victorious,) that ten died of their wounds on the day of battle; and, from what has been experienced on similar occasions, we may reasonably infer, that some cases of the kind alluded to above, were among the number; but I trust I shall be pardoned the remark upon conjecture only, as I am not in possession of any authentic facts; but, reading the statement, the newspapers made a deep impression, and called to my mind the present remarks, which I am now thus candidly submitting to the public, and which I am confident will, upon minute investigation, appear but too extensively founded in fact; whilst, in great hospitals, in private practice, or any where else but under the influence which naval engagements excite, such fatal occurrences are extremely rare; for, in the principal naval hospitals, in the public hospitals of London, and other great cities where amputations are so frequently performed, and also in private practice, where operations are performed under a tranquil and regular state of the system, fatal results of this kind, during the amputation of a limb, or ensuing from it as an immediate cause, are extremely uncommon. But in the peculiar situation of the patient we are now considering, especially after he has been long long fatigued in battle, fatal results of this nature will be found very frequently to have taken place. The cause of such a disastrous event under the circumstances I have mentioned, cannot be ascribed to any error in the operation, or immediate mismanagement on the part of the surgeon, but to a general law of the animal system, the effects of which, at this particular juncture, was not foreseen, but which in the sequel so impressively merits our particular attention; for I may venture to say it is an event the young practitioner is altogether unprepared for, and the scene is closed before much time is allowed for reflection; but, when once put upon his guard, it is an event, I think, he may, in very many instances, be able to avert.

During the usual preparations for battle on-board a man of war, much excitement both of body and mind must take place. The subsequent scene of action, by laboring at the gun perhaps for some hours, still more eminently induces excitement of the vascular and organic systems; but, upon a cessation of those exciting causes, after having been so long in force, and the infliction of so extensive an injury from a cannon-ball, a very great exhaustion of the sensorial power must follow. The degree of exhaustion being proportionate to the stimulus previously applied, we need not, therefore, be long at a loss in assigning a powerful cause for the fatal events we have above enumerated.

From this degree of sudden exhaustion, a state of the vascular system takes place, forcibly analogous to the cold stage of fever. An exceeding quick and small pulse, with great diminution of strength, an evidently feeble action of the cutaneous and capillary vessels, are symptoms which naturally follow in this state, upon such a severe wound being inflicted, which, together with the subsequent operation, must have powerful effects. The immediate collapse of the vascular system which thus takes place, is too great for the heart and arteries to overcome, and the unfortunate warrior perishes, we may say, in a state of syncope. Perhaps the vitiated air which they respire in this state, when brought below to the cockpit, may prove an auxiliary cause; but that is rather a difficult circumstance to obviate. The procuring a constant accession of fresh air from without, at this period, is in a certain degree impossible; the only remedy we can substitute, is diffusing the nitrous vapor by means of the fumigating materials furnished to the navy; but even that, though of importance, merits only a secondary consideration: the great object to be kept in view, is to support the energy of the system, which is here so suddenly and so powerfully abstracted. Stimulants received by the sto-

 mach mach must become our immediate resource, and wine certainly presents itself on this occasion as the most salutary stimulus we can make use of, but it must be administered with a prompt and bold hand, and that for some time before the operation is performed, for the occasion admits of very little time for reflection. Could the wine be made warm, it would have a more immediate effect; or, when the sinking state of the patient proves indicative of higher stimulants being required, a portion of ginger powder, black pepper, or any other stimulating aromatic, can be added to the wine, and which would answer better, and with more immediate effect, than if we were to administer spirituous or volatile stimulants. The state of the pulse will chiefly direct us; the pulsations may be exceedingly quick, but then they will be felt small and feeble. It is an experienced fact, that when the system is laboring under a state of much debility or exhaustion, the pulse will be found extremely frequent, though small and weak; but, upon a due portion of invigorating stimulants being administered, such as wine, &c. the pulse becomes less frequent, full, and soft. The remedy may be then said to have answered our hopes. The use of wine on those occasions has been, I believe, in general most strenuously withheld, under the idea of its exciting hæmorrhage, but certainly most erroneously; a wounded artery will bleed whilst the patient lives, whether he has taken any wine or not; and, allowing that the disposition to hæmorrhage may be a little increased by it, yet this is a reason for withholding it by no means equal to that for which I plead the exhibition. From what has been already advanced, it may be proposed to defer any operation that may be requisite until the system has recovered its wonted tone and action. This might prove advantageous were it practicable; but we cannot trust a wounded limb, without a tourniquet screwed upon it, and the instrument, applied so as to interrupt the circulation in the part, must soon excite much swelling and inflammation of the limb; and the mischief arising from operating upon inflamed and tumefied muscular substance, is but too obvious to require any comment. The limb ought certainly to be removed before any symptom of this kind takes place.

I have thus, in a cursory manner, endeavored to detail what actual observation has taught me, aided by authentic information which I have had from others of still more experience upon this most important point of practice, which I sincerely hope may afford an useful hint to those gentlemen who have not yet experienced such scenes, but who ere long may have the lives of so many of those valuable men placed placed in their hands. They must be well aware how precious that moment is, and must therefore act with firmness and promptitude. Their avocations of duty on the day of battle are certainly extensive, but much may be done by timely and judicious exertion. And I must beg leave in this place to call to remembrance a hint which has already been inculcated through the medium of this publication, i. e. to bring the cutting instruments to a warm temperature, by immersing them in warm water previous to being used. It is a hint which cannot be too often repeated; here it is practicable, and here it will prove eminently beneficial. Whilst the surgeon and his assistants are more importantly employed, the chaplain and purser (who are here joined in duty with him) being gentlemen possessing enlightened and discriminating minds, will be able with a transient direction to administer wine, and other invigorating stimulants, to the exhausted and sinking wounded patients who have now come under his sole care; means which he may direct to be made use of freely, as occasion requires. It is chiefly in injuries which affect the brain, extensive contusions, or wounds where surgical means cannot reach, that such stimulating remedies become inadmissible; but in most other cases where any portion of the body is to be removed by operation, they can freely be administered, without much apprehension of great arterial excitement, or inflammatory diathesis being induced, as we have it so completely in our power to obviate any subsequent inflammatory tendency which may prevail; I am therefore conscious, that, by the prompt and free exhibition of the means we have recommended, at this peculiar juncture, many valuable lives may be preserved, which would otherwise be doomed to perish.

I have the honor to be, Gentlemen,

Your most obedient and humble Servant,

JAMES LITLE, Surgeon,

*His Majesty's Ship Salvador del Mundo,*
*at Plymouth, June* 5, 1812.

